# Influence of Interleukin-1 Beta on Platelet-Poor Plasma Clot Formation: A Potential Impact on Early Bone Healing

**DOI:** 10.1371/journal.pone.0149775

**Published:** 2016-02-24

**Authors:** Xin Wang, Yan Luo, Paul P. Masci, Ross Crawford, Yin Xiao

**Affiliations:** 1 Department of Spine, Affiliated Hospital of Zunyi Medical College, Zunyi, Guizhou Province, China; 2 Institute of Health and Biomedical Innovation, Queensland University of Technology, Brisbane, Queensland, Australia; 3 Australia-China Centre for Tissue Engineering and Regenerative Medicine, Queensland University of Technology, Brisbane, Queensland, Australia; 4 Translational Research Institute, School of Medicine, The University of Queensland, Brisbane, Queensland, Australia; University Hospital Medical Centre, GERMANY

## Abstract

**Objectives:**

Hematoma quality (especially the fibrin matrix) plays an important role in the bone healing process. Here, we investigated the effect of interleukin-1 beta (IL-1β) on fibrin clot formation from platelet-poor plasma (PPP).

**Methods:**

Five-milliliter of rat whole-blood samples were collected from the hepatic portal vein. All blood samples were firstly standardized via a thrombelastograph (TEG), blood cell count, and the measurement of fibrinogen concentration. PPP was prepared by collecting the top two-fifths of the plasma after centrifugation under 400 × *g* for 10 min at 20°C. The effects of IL-1β cytokines on artificial fibrin clot formation from PPP solutions were determined by scanning electronic microscopy (SEM), confocal microscopy (CM), turbidity, and clot lysis assays.

**Results:**

The lag time for protofibril formation was markedly shortened in the IL-1β treatment groups (243.8 ± 76.85 in the 50 pg/mL of IL-1β and 97.5 ± 19.36 in the 500 pg/mL of IL-1β) compared to the control group without IL-1β (543.8 ± 205.8). Maximal turbidity was observed in the control group. IL-1β (500 pg/mL) treatment significantly decreased fiber diameters resulting in smaller pore sizes and increased density of the fibrin clot structure formed from PPP (*P* < 0.05). The clot lysis assay revealed that 500 pg/mL IL-1β induced a lower susceptibility to dissolution due to the formation of thinner and denser fibers.

**Conclusion:**

IL-1β can significantly influence PPP fibrin clot structure, which may affect the early bone healing process.

## Introduction

Fracture hematoma (blood-clot) that is formed immediately after injury is suggested to play an important role in fracture union, because the removal of a blood-clot during operative stabilization can impair the initial phase of healing [1]. Fracture healing is a unique physiologic process characterized by three overlapping stages: fracture hematoma formation and the initial inflammatory response, callus formation, and early bony union and bone remodeling [2]. Although numerous studies have focused on bone biology and fracture healing, to our knowledge, little reported literature is available on early bone healing, such as studies characterizing the fracture hematoma fibrin network and the factors that impact fibrin clot quality. Hemostasis (blood coagulation) is initiated by platelets; however, stabilized fibrin formation throughout hematoma development involves several other components, such as immune cells and inflammation cytokines [3, 4]. Therefore, the importance of hematoma at a fracture site is increasingly being recognized for its supportive role in providing a transient fibrin matrix to allow cell infiltration, proliferation, and differentiation, as well as serving as a short-term ‘reservoir’ for growth factors released from activated platelets and adjacent tissues [5].

To better facilitate biocompatibility for bone regeneration, a wide range of autologous blood products, such as platelet-rich plasma (PRP) and platelet-rich fibrin (PRF), have been employed in a clinical setting [6–8]. Indeed, PRP or PRF is a fraction of plasma enriched with activated platelets connected with fiber filaments, serving as a functional source of growth factors [9]. However, the use of PRP to stimulate new bone regeneration has been controversial, owing to its rapid release of growth factors from the fibrin network [10, 11]. A more reasonable assumption for PRP not being suitable for bone-defect healing is its relatively denser fibrin network that impedes the cellular infiltration from surrounding tissues [12, 13]. Alternatively, artificial fibrin scaffolds with a small pore size, formed by the extensive application thrombin, have also been reported to delay natural healing process in a rat model [14], indicating that fibrin structure alterations (fiber diameter, density, pore size, porosity, branch points, and branch junctions) can considerably influence the bone healing process. It has been revealed that hematomas composed of loosely-woven fibrin structure with thicker fibers can better expedite the egress of mesenchymal stem cells (MSCs) and endothelial cells into injured sites, diffusion of oxygen and nutrients, and removal of metabolic waste [15, 16].

Acute phase response (APR) is the earliest response to vascular injury at fracture sites, characterized by the generation of acute phase proteins, such as fibrinogen and cytokines [17]. After vascular compromise, activation of blood coagulation occurs quickly when whole blood interacts with the surface of broken bone fragments. The adsorption of plasma proteins is deemed to initiate platelet reaction and an extrinsic coagulation cascade, resulting in thrombin enzyme and fibrin formation, and finally a hematoma at the fracture sites [18]. Currently, a growing body of evidence indicates that inflammation and hematoma formation are closely intertwined [3, 4]. It has been demonstrated that IL-1β, a major inflammatory cytokine released from activated platelets and immune cells, can bind to fibrinogen thus preserving its activity [19], but its impacts on fibrin clot structure has garnered little attention. Notably, the expression of IL-1 beta, especially with the simulation of lipopolysaccharides (LPS), has been documented in early bone fracture healing (day 3) [20]. Studies with mice tibial fractures showed that the local administration of IL-1β exerted an inhibitory effect on proliferation of MSCs [21], and IL-1β antagonist can facilitate fracture restoration in rat model [22]. While most investigators used the dose of IL-1β ranging from 10 pg/mL to 1000 pg/mL [23], our recent study (unpublished data) also revealed that the levels of IL-1β were significantly higher in delayed bone healing defects (745.40 ± 99.19 pg/mL) than ones in natural bone healing defects (57.46 ± 4.72 pg/mL). Therefore, we selected 50 and 500 pg/mL as experimental concentrations to observe whether IL-1β is a major determinant of altering clot construct, thus influencing the healing process of large bone defects.

PPP is the supernatant of plasma with low amounts of platelets and blood cells, which contains elevated levels of fibrinogen to generate a non-turbid fibrin-rich clot once activated [24]. Moreover, fibrin clot formed from PPP could be used as an autologous degradable scaffold for tissue engineering [25]. Exempt from disturbances of other blood plasma components (platelets and blood cells), PPP on behalf of whole blood is often used to investigate the inherent architecture of fibrin clots [26, 27]. Therefore, this study aimed to determine whether IL-1β could influence the fibrin structural properties in PPP clots.

## Materials and methods

### 2–1 Reagents

Human Alpha Thrombin (HT 1002a) and human plasminogen (HPg 2001) were acquired from the Enzyme Research laboratories (Bulimba, Australia). Human Fibrinogen (Oregon Green^™^ 488 Conjugate) and Interleukin-1β were purchased from Invitrogen (Victoria, Australia). Recombinant tissue-type plasminogen activator and d-Dimer (d2d) ELISA Kit were ordered from antibodies-online Inc. (Atlanta, United States).

### 2–2 Standardization of blood sampling

Blood was preserved by collecting 900 μL of blood from the hepatic portal vein of Fisher rats in 100 μL of 4% tri-sodium citrate (9:1). Blood samples were mildly inverted 6 times, and kept upright for at least 30 min. For the TEG test, 320 μL of blood was transferred gently into a disposable plastic TEG cup (Haemoscope Corporation) containing 20 μL of 0.2 M CaCl_2_ solution, and the assay was performed on a TEG^®^ 5000 Series Haemostasis Analyser at 37°C within 1 h of blood collection. The parameters were automatically traced with TEG, including reaction time (R, seconds), coagulation time (K, seconds), angle (α, degrees), and maximum amplitude (MA, mm). Details of this method are described in a previous report [28].

Two-hundred microliters of citrated rat whole blood volume was analyzed with the Haematology Analyser (XT-2000i) to measure red blood cell (erythrocyte), white blood cell (leukocyte), and platelet populations. Additionally, 2 mL of citrated blood volume was centrifuged at 10,000 rpm (1300 × *g*) for 15 min at room temperature. Then, a 100 μL aliquot of the supernatant was transferred to a 1.5 mL plastic tube for analysis using the ACL TOP CTS Haemostasis Analyser, which detected the fibrinogen concentration in the plasma.

### 2–3 PPP preparation

PPP was prepared according to previously published protocols [8, 29]. Briefly, 5 mL of blood drawn from hepatic portal vein were added to 15 mL Falcon tubes. The fresh blood samples were immediately transferred into a centrifuge. After the centrifugation at 3000 rpm (400 × *g*) for 10 min at 20°C, the mixture was divided into three layers: the upper layer was acellular plasma (PPP), the lower layer was red blood cells (RBC), and the middle layer was a buffy coat layer (PRF) that was rich in platelets and had a paucity of leucocytes. PPP was completely decanted and stored at -80°C. Fibrinogen concentration in PPP solutions was measured by the ACL TOP CTS Haemostasis Analyser and 1 mg/mL was used in this study.

### 2–4 Turbidity

Fibrin polymerization in PPP clots was monitored in a 96-Well MicroWell Plate (Thermo Scientific, USA) by tracing alterations in turbidity at 405 nm (A_405_) every 15 s for 50 min, at 37°C using a Microplate Absorbance Reader (Bio-Rad X-Mark spectrophotometer) as described previously [30, 31]. The 100 μL PPP solution (1 mg/mL) with IL-1β (0, 50, or 500 pg/mL) in HEPES buffer (20 mM HEPES, 150 mM Nacl, pH = 7.4) was preincubated for 10 min at 37°C. The PPP solution mixed with only HEPES buffer instead of IL-1β was defined as the control. Additionally, thrombin (0.1 U/mL) and CaCl_2_ (10 mM) were blended and preincubated for 10 min at 37°C. The lag time represented the rate of protofibril formation and the size and number of fibers were typified by the maximal turbidity.

### 2–5 Scanning electron microscopy (SEM)

SEM studies for blood clot characterization was carried out as described previously [32, 33] with the following minor modifications. PPP clots were formed by the PPP (1 mg/mL) with IL-1β (0, 50, or 500 pg/mL), thrombin (1 U/mL), and CaCl_2_ (10 mM) in HEPES buffer. The PPP solution in HEPES buffer without IL-1β mixed with thrombin and CaCl_2_ was defined as the control. After 2 h at room temperature, clots were rinsed with phosphate buffer saline (PBS) (pH = 7.4) at least 3 times, and then fixed in 3% glutaraldehyde overnight. Thereafter, clots were transferred to a cacodylate buffer (0.1 M), post-fixed with 4% osmium, and dehydrated with an ethanol gradient. Specimens were mounted on carbon tabs, and sputter coated with gold-palladium. All specimens were analysed under a Zeiss SEM (FEI, USA) at a magnification of 10,000 ×, which could detect an individual fiber. Fiber structural parameters (thickness and density) were further measured using the Image J software (version 1.43) according to a modified method of Undas et al [34]. For quantitative analysis of the pore sizes in fibrin clots, the thresholding algorithm (0, 60) was run to highlight the black areas (no fibres). The black (space) portions of the images were quantified and defined as 2-D percentage porosity. Pore area was obtained using the performing particle analysis function in Image J [35, 36].

### 2–6 Confocal microscopy (CM)

PPP solutions (100 μL, 1 mg/mL) with IL-1β (0, 50, or 500 pg/mL) in HEPES buffer was coupled to the Oregon Green^™^488 fibrinogen (0.1 mg/mL) on the coverslips (Thuringowa, Australia). After the addition of thrombin (1 U/mL) and CaCl_2_ (10 mM), PPP clots were formed in a moist atmosphere at room temperature overnight. Clots were prepared for observation using a Nikon A1R Confocal Microscope with a 40 × 1.3 NA oil objective as described previously [30, 37] with following modifications. The scans were taken using the 6 × zoom-in mode. After acquisition of PPP clot morphological images, maximum intensity projections (MIP) images were analyzed. Fiber size was measured by drawing a perpendicular line across the fiber, avoiding any junctions. Moreover, the fiber density was counted by drawing a 50-μm line across the middle transections of the scanned MIP image. The representative morphologies of fibrin clot were captured.

### 2–7 Clot lysis assay

Clot lysis is strongly correlated with fiber thickness and density [24]. The effect of various concentrations of IL-1β on overall clot degradability was evaluated and its association with the fibrin architecture was investigated. The fibrinolytic process was evaluated by detecting the amounts of fibrin degradation product (d-dimer) when the fibrin clots were dissolved by the plasmin, which yielded activation of plasminogen in the presence of tissue-type plasminogen activator.

A suspended clot system was applied in this study as described previously [33, 38]. PPP solutions (100 μL, 1 mg/mL) with IL-1β (0, 50, or 500 pg/mL) in HEPES buffer, thrombin (1 U/mL), and CaCl_2_ (10 mM), were simultaneously added to the vials for 2 h at 37°C to allow complete clot formation. Clots were transferred into the new vials and suspended in 3 mL of PBS buffer containing human plasminogen (Glu-plasminogen, 5.4 μg/mL final concentration; American antibodies-online Inc., USA). The clot lysis process was initiated by adding tissue plasminogen activator (tPA, 0.25 μg/mL final concentration; Australia Enzyme Research laboratories Inc., AU) at 37°C with gentle agitation. Aliquots of the supernatant (100 μL) were pipetted at timed intervals (1, 4, 8, 18, and 24 h) and centrifuged at 1000× *g* for 3 min for d-dimer detection. The extent of clot lysis was detected by measuring the amounts of d-dimer by use of a d-dimer ELISA kit (American antibodies-online Inc., USA). In addition, weight losses of clots were also recorded at the indicated time points.

### 2–8 Ethics statement

Ethic approval was granted for this study from Queensland University of Technology (approval number: 1400000023).

### 2–9 Statistical analyses

Results were represented as the means ± standard derivation. For statistical evaluation, Student’s *t*-test was used for two group differences and one-way analysis of variance (ANOVA) was used for three group differences, followed by post-hoc test. A p value <0.05 was considered statistically significant.

## Results

### 3–1 Standardization of blood collection using hematological parameters by the analysis of TEG, Haematology, and Haemostasis Analyser

**Fig 1:**
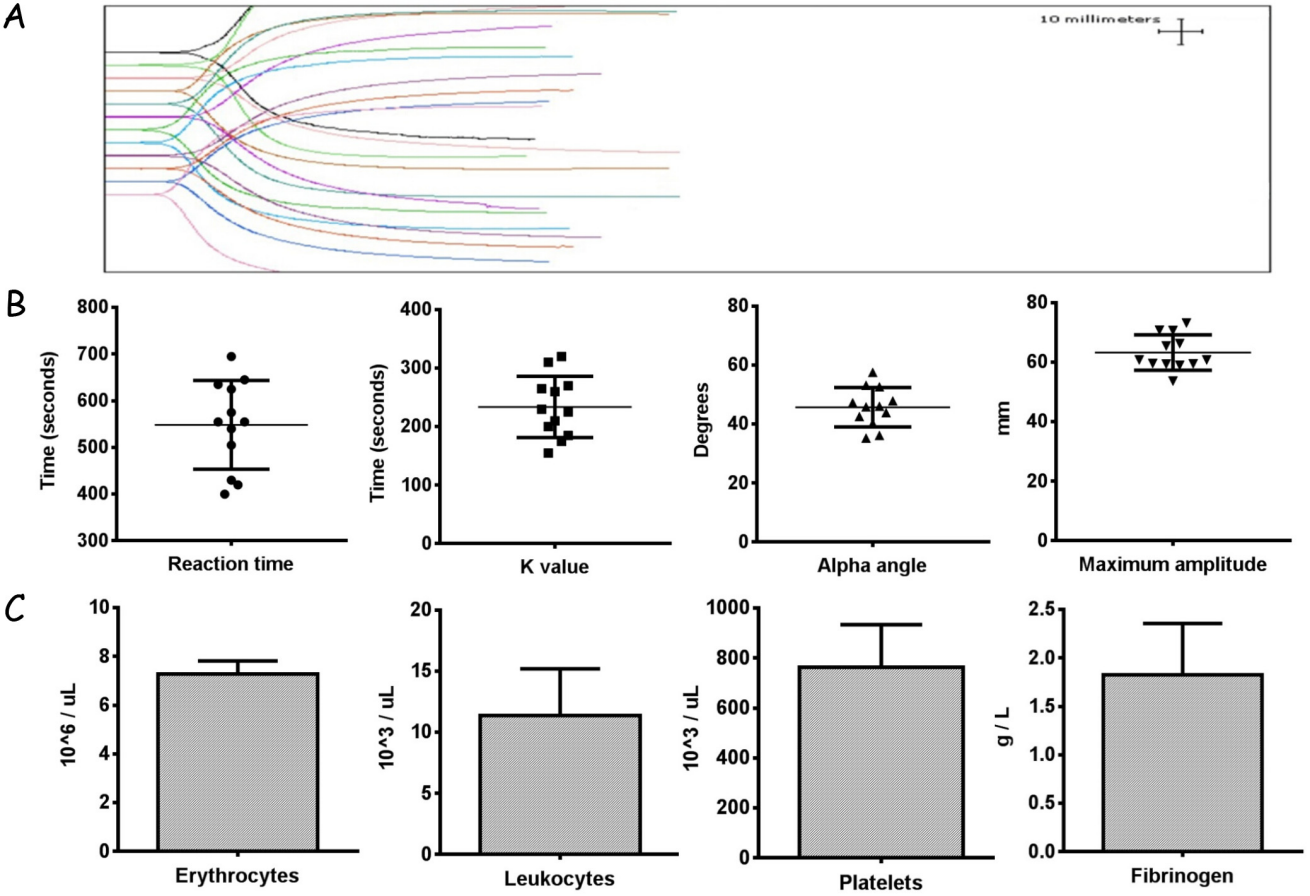
Profile of TEG parameters (A, B), blood cell count, and fibrinogen concentration (C). This profile comprised two processes, namely, thrombosis and fibrinolysis. Thrombosis was described by four important parameters: R time; which was calculated from the time that the blood was pipetted into the TEG analyzer till initial fibrin clot formation. K time was the time at which clot formation reached amplitude of 20 mm, representing clot formation speed. In addition, α angle denoted the level of fibrinogen. MA value was a reflection of platelet function and aggregation, which indicated that clot strength (stiffness) reached the maximum amplitude.

From panel A in Fig 1, the TEG parameters from the collected blood were detailed as follows: mean reaction time (R, 548.3 ± 95.09, s), mean coagulation time (K, 233.8 ± 52.27, s), angle value (α, 45.8 ± 6.71, degrees), and mean maximum amplitude (MA, 63.3 ± 5.9, mm). In panel B and C, the outcomes of hematology parameters were consistent with the described literature using Haematology and Haemostasis Analyser measurements [28, 39, 40]. The performance of TEG was aimed to standardize the constituents of whole blood within the biological range, which minimized blood sample variation between individuals and ensures that PPP solution made from whole blood was at an identical level.

### 3–2 Fibrin polymerization

**Fig 2:**
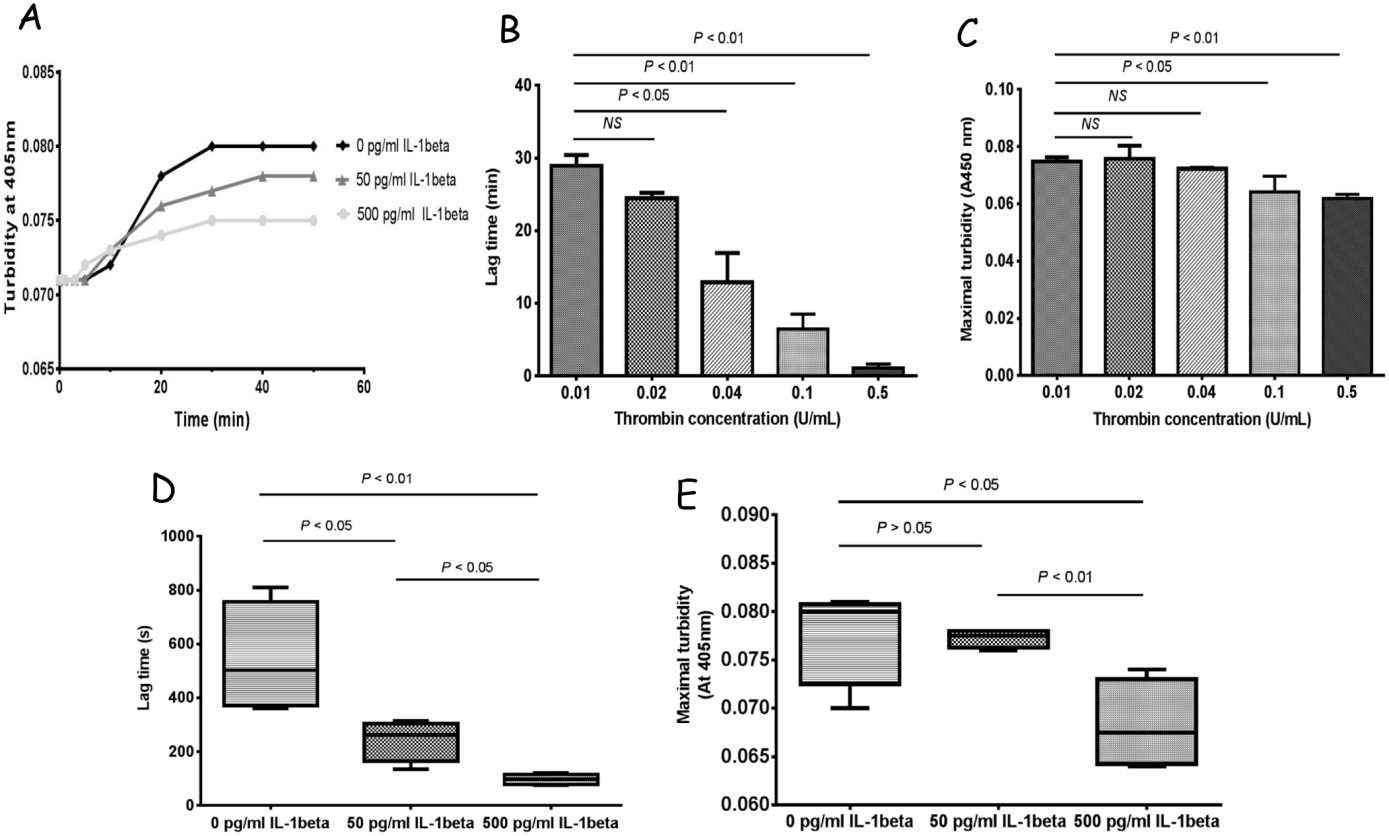
The effect of IL-1β on fibrin polymerization of PPP clots. Effect of IL-1β on polymerization of PPP clots (A, D, E) and effect of different concentrations of thrombin on PPP clots (B, C). In figure B and C show the lag time when turbidity absorbance starts to rise to 0.001 and maximal turbidity in PPP solutions with thrombin (0.01, 0.02, 0.04, 0.1 or 0.5 U/mL) and CaCl_2_ (10 mM) in HEPES buffer (pH = 7.4). In figure A, fibrin polymerization was plotted using three different colors: dark (control group), dark gray (50 pg/mL IL-1β group), and light gray (500 pg/mL IL-1β group). Figure D and E represent lag time and maximal turbidity in PPP solutions by addition of thrombin (0.1 U/mL) and CaCl_2_ (10 mM), respectively. Data from 5 replicates were analyzed by unpaired Student *t*-tests. NS indicated no significant differences.

Fig 2 showed that variations of thrombin concentration (more than 0.1 U/mL) can significantly affect lag time and maximal turbidity during PPP clot polymerization process compared with control (0.01 U/mL) (*P* < 0.01) (B, C), suggesting that clot kinetics (lag time and maximal turbidity) were inversely proportional to increases in thrombin concentrations. Furthermore, the dynamic of fibrin polymerization in PPP solutions was characterized by measurement of turbidity curves by the addition of thrombin (0.1 U/mL) and CaCl_2_ (10 mM) with or without IL-1β (A). Lag time was detected from the turbidity curve to be 543.8 ± 205.8 s in control groups, which was significantly longer than the IL-1β groups (D) (243.8 ± 76.85 s in the 50 pg/mL and 97.5 ± 19.36 s in the 500 pg/mL) (*P* < 0.01). Maximal turbidity, the final optical density, was determined from the turbidity curve to be 0.0778 ± 0.0052 in control group, while it was 0.0773 ± 0.001 in the 50 pg/mL groups and 0.0683 ± 0.0023 in the 500 pg/mL groups (E) (*P* < 0.05).

### 3–3 Fibrin architecture

**Fig 3:**
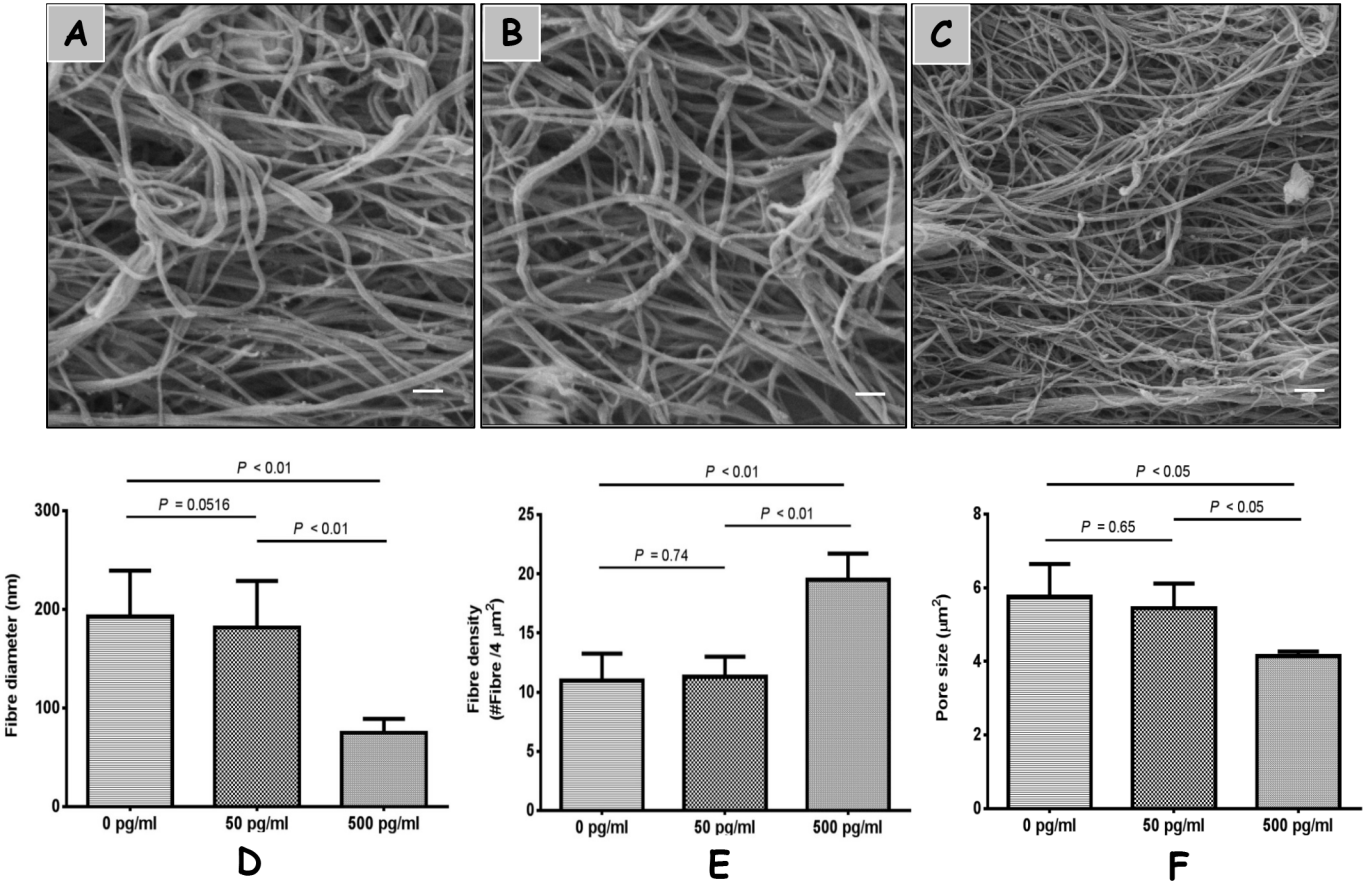
The effect of IL-1β on fibrin architecture of PPP clots. Morphology of PPP clots (*n* = 3) architecture (A, B, C) formed by different concentrations of IL-1β were analyzed using Zeiss SEM. Fiber diameter (D) (*n* = 150), density (E), and pore size (F) were measured using the Image J software. Fibers appear significantly different in the IL-1β groups at the concentration of 500 pg/mL with a thinner diameter, denser, and lower porosity (scale bar = 2 μm).

As evident from the graphs shown in Fig 3, PPP clots formed in the absence of IL-1β (A) or in the presence of IL-1β (B, C) revealed a multitude of individual fibres in the fibrin networks. The mean diameters of fibers in PPP clots were 192.6 ± 46.84 nm in the control groups, and 181.6 ± 47.40 nm in the 50 pg/mL IL-1β groups (D). Fiber diameters decreased dramatically in the 500 pg/mL IL-1β treatment group with fiber diameters ranging 74.70 ± 14.27 nm (D) (*P*<0.01). In contrast, there was a higher density of fibers in the 500 pg/mLIL-1β groups (19.50 ± 2.22) compared to the other groups (E) (11.00 ± 2.26 and 11.30 ± 1.70 in the control groups and 50 pg/mL IL-1β groups, respectively). Mean pore areas of 500 pg/mL IL-1β group were significantly (*P*<0.05) smaller (range, 4.053 to 4.289 μm^2^) than that of the control groups (range, 5.219 to 6.787 μm^2^) and the 50 pg/mL IL-1β groups (range, 4.854 to 6.172 μm^2^) (F).

### 3–4 Fibrin clot structure

**Fig 4:**
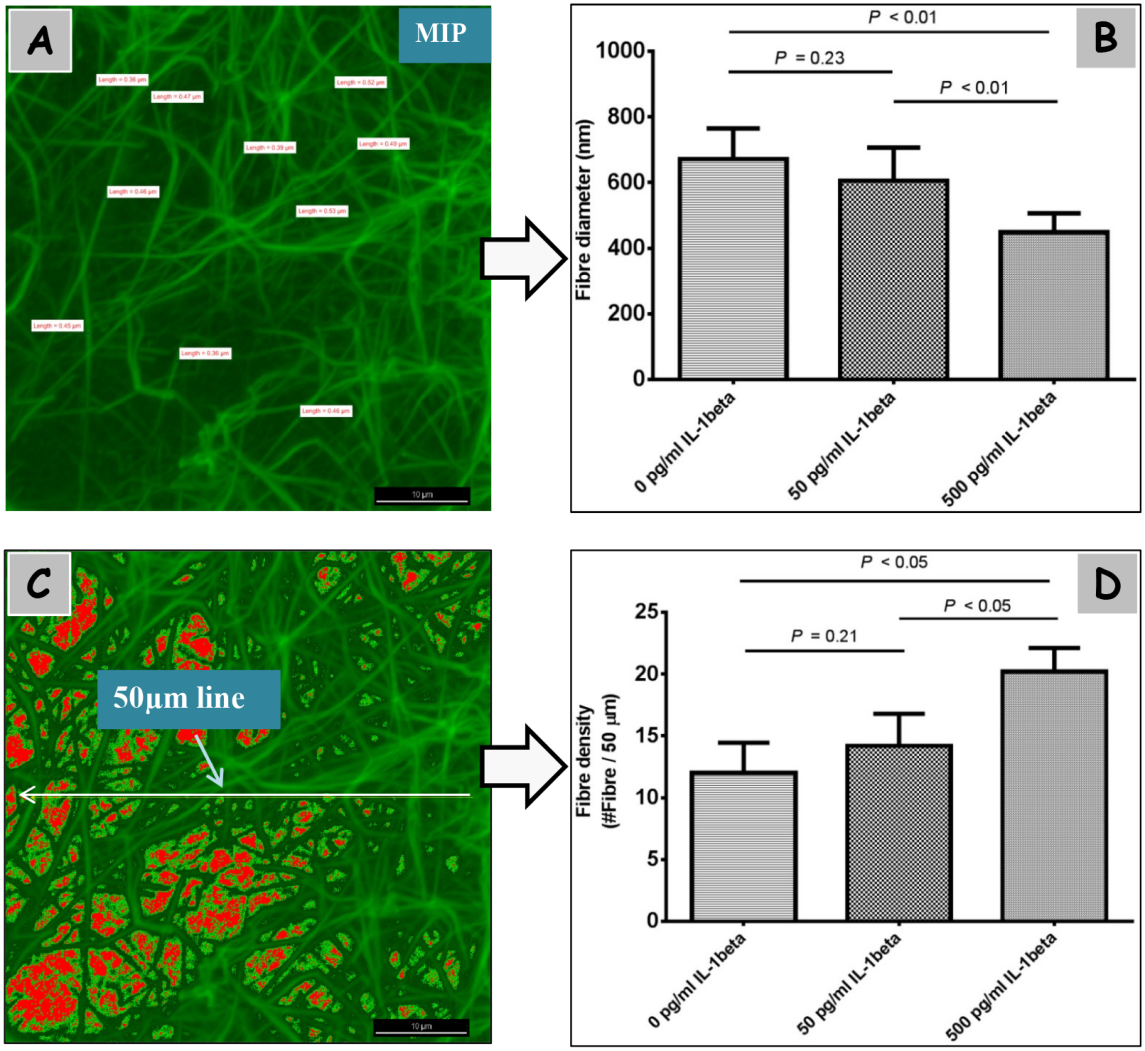
The effect of IL-1β on fibrin clot structure of PPP clots. Structural morphology of PPP clots formed by 500 pg/mL IL-1β (*n* = 3) (A, C). In 0, 50, 500 pg/mL IL-1β groups, using the function of Annotations and Measurements, fiber diameters were determined from maximum intensity projections (MIP) (B). Fiber densities were counted in the Intensity graph obtained by calculating the peaks (the number of fibers) that cross a line of 50 μm (D). Scale bars represent 10 μm.

The results in Fig 4 indicated that the denser fibrin structure with thinner fibers was formed in the 500 pg/mL IL-1β groups. The oversaturated color and noise were minimized by the application of look-up tables (LUTs), and, consequently, the measurements of fiber parameters would be more reliable. Fiber diameters were 671.4 ± 93.71 nm in the control groups and 605.7 ± 100.8 nm in the 50 pg/mL IL-1β groups. However, fiber widths were merely 448.6 ± 57.57 nm in the 500 pg/mL IL-1β groups. Using the Intensity Profile and Object Count tools, the amounts of fibers were found to be 12.00 ± 2.45,14.20 ± 2.59,20.20 ± 1.92 in the 0, 50, and 500 pg/mL IL-1β groups, respectively.

### 3–5 Fibrinolytic assay

**Fig 5:**
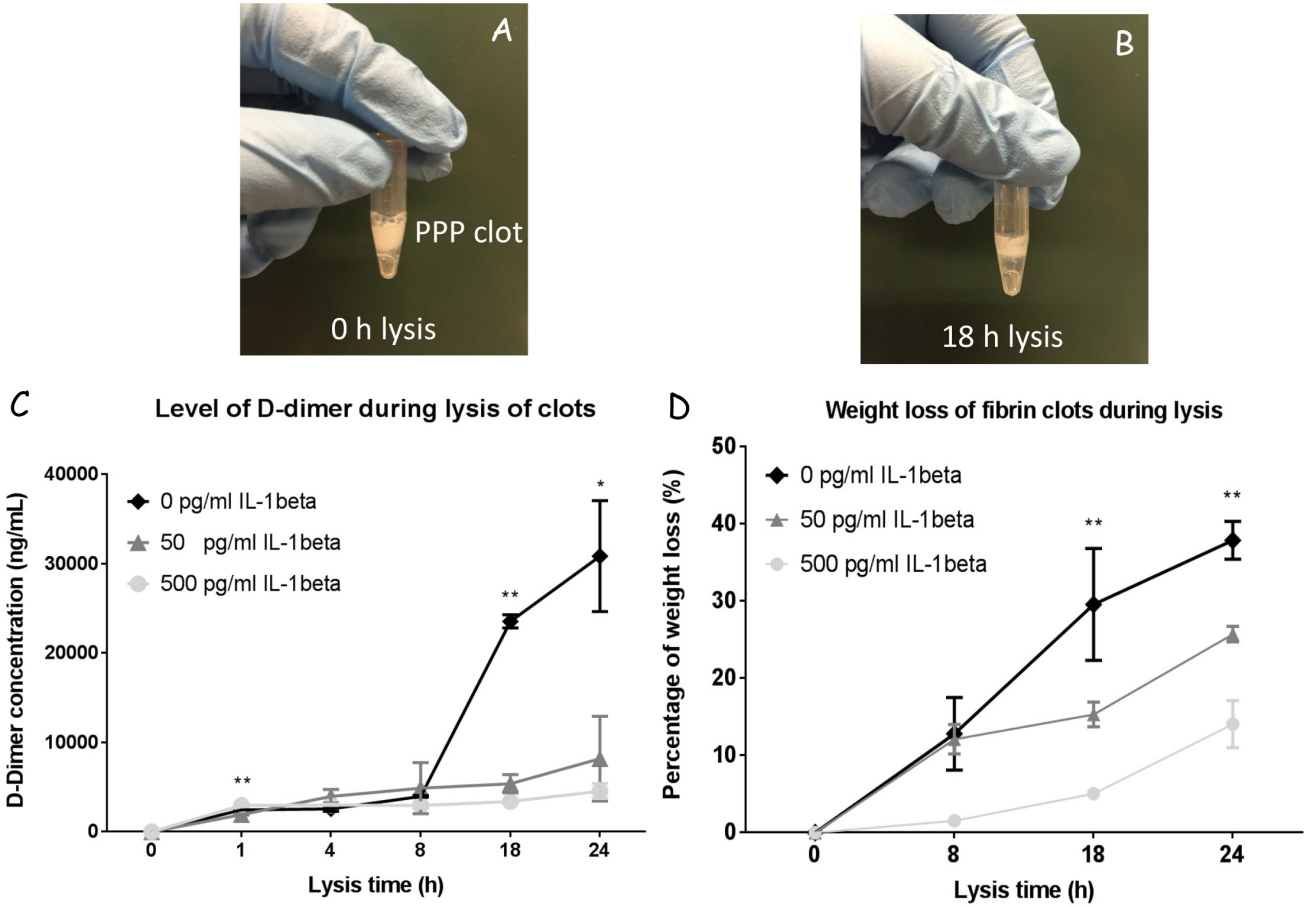
The effect of IL-1β on fibrinolytic activity of PPP clots. A PPP clot formed by the addition of thrombin (1 U/mL) and CaCl_2_ (10 mM) for 2 h at 37°C (A), after 18 h of lysis, the size of the clot showed a notable decrease (B). Releases of d-dimer and weight loss were measured during PPP clot lysis over 24 h (C, D). The d-dimer levels of clots formed by various concentrations of IL-1β (C) and the percentage of weight losses denoting the lysis of clots (D). * P ≤ 0.05, **P ≤ 0.01.

To evaluate the effect of various concentrations of IL-1β on fibrinolysis, d-dimer levels and weigh losses from the clots were compared in one-way ANOVA (Fig 5). At 1 h, all clots revealed significant increase of d-dimer, reflecting the fibrinolytic activity has commenced. The 50 pg/mL IL-1β groups initially underwent a relatively slower rate of fibrinolysis (*P* < 0.05). However, no significant differences were found at the following 4 and 8 h. At 18 and 24 h, the concentrations of d-dimer rapidly increased in the control groups compared to IL-1β groups, suggesting that fibrin fibers in the control group were subjected to dissolution. This was also in line with the outcomes from weight losses among the three groups.

## Discussion

The prevalence of complications from bone fracture treatments for large bone defects, such as delayed bone union and bony non-union, continue to be clinical challenges. Although an accumulating number of traditional approaches, such as bone substitutes, have been introduced in implantology, the use of these therapies is often unpredictable and complicated with potential safety issues, such as a foreign body reaction, which leads to fibrotic encapsulation and implant dysfunction [41, 42]. When vascular injury occurs at a fracture site, platelets and the fibrin-producing clotting system, which mediate hemostasis, produce a functional fracture hematoma that seals the bone broken fragments and re-constructs vascular continuity [43]. While platelet aggregation provides temporary closure of the defect, the initiation of a multistep coagulation reaction in a highly efficient manner ensures that it remains mechanically stable via generation of the glue-like fibrin within a growing fracture hematoma [44]. Parallel with the recruitment of platelets, a large number of platelet-related molecules, such as, fibrinogen, growth factors, and pro-inflammatory cytokines, are produced or activated, thereby participating in fibrin generation [45]. During the dynamic process of fibrin polymerization, the concomitant activation of blood coagulation and inflammation has been suggested to be closely linked [46]. It is well-known that the fibrin structure determines cell migration and proliferation during the healing process.

In this study, in order to maintain consistency among blood samples collected from rats, all samples were first subjected to standardization using the TEG, Haematology, and haemostasis analyser. By means of the formulation of artificial PPP clots *in vitro*, we observed from turbidity measurements that the lag time (97.5 ± 19.36 s) in the 500 pg/mL IL-1β group was markedly shortened compared with the control groups (543.8 ± 205.8 s; *P* < 0.01). This indicated that the single application of IL-1β (500 pg/mL) can yield the formation of PPP clots with high density of fibers, possibly owing to expedition of the γ-γ crosslinking between protofibrils [47]. Accordingly, there was a remarkable decrease in maximal turbidity in the 500 pg/mL IL-1β groups, implying that the fiber size became thinner than the control groups (*P* < 0.05). Furthermore, morphological parameters of the fibrin fiber (diameter and density) were characterized using SEM and CM, which implied that IL-1β (500 pg/mL) with thrombin could dramatically alter the structural properties of artificial fibrin clots (*P* < 0.01). IL-1β affects fiber size, possibly by directly affecting the carboxyl-terminal site of the γ chain in fibrinogen [48], which has a close relation to the γ-γ crosslinking in fibrin polymerization [47, 49].

Clot fibrinolytic activity using a suspended clot system revealed that the IL-1β groups initially experienced a relatively slower rate of fibrinolysis according to the detection of d-dimer amounts and weight loss. At 18 h of clot lysis, the d-dimer concentration significantly increased in the control groups compared to IL-1β groups, which was in accordance with the loose and thicker clot network observed in the control groups under SEM and CM. This is further evidenced by the literature, which shows that fibrinolysis occurs much faster on a loose and thicker fibrin network rather than on a tight and thinner one [50, 51].

The main limitation of this study is that a PPP clot does not completely stand for a whole blood clot (hematomas). However, the advantage of using PPP is that exposure of individual in the clots fibrin fiber becomes evident for characterization under observations of SEM and CM. Currently, to our best knowledge, few literature reporting how to detect and measure fibrin parameters of whole blood clots under CM. Given that thrombin produced by remaining platelets in PPP solutions is insufficient to initiate coagulation cascade, addition of exogenous thrombin is requisite for PPP clot formation as described by Gersh et al [27] and Talens et al [52].

Fracture hematoma is widely believed to be a biologically active tissue where thrombin, the major end product of the coagulation cascade, is activated in the event of an injury to hemostasis. In addition, a diverse subset of other cellular and molecular elements including proinflammatory cytokines is also recognized as being essential to exploit their procoagulant repertoire, thereby propagating coagulation [53]. The clarification of specific underlying relationship between proinflammatory cytokines, such as IL-1β and fiber structure, in this study expands our understanding of its thrombogenesis, and should consequently facilitate development of a novel strategy for treatment of large bone defect via alteration of fiber structures in hematomas.

## Conclusion

In summary, this study confirmed that by simply varying the IL-1β concentration in a PPP solution, the lag time of protofibril formation and maximum turbidity without additional fibrinogen or thrombin can be significantly reduced. Therefore, controlling proinflammatory cytokines such as IL-1β concentration may present a novel intervention for tailoring fibrin clot architecture to improve the repair and regeneration of skeletal tissues.
